# Unveiled: A Case of N-Methyl-D-Aspartate Receptor Antibody Encephalitis With Delayed Diagnosis of Ovarian Teratoma

**DOI:** 10.7759/cureus.54486

**Published:** 2024-02-19

**Authors:** Patricia Fleur J Andaya, Alejandro Bimbo F Diaz

**Affiliations:** 1 Neurology, St. Luke's Medical Center, Quezon City, PHL

**Keywords:** autoimmune encephalitis, delayed teratoma, seizures, dyskinesia, behavioral changes, nmda-receptor encephalitis

## Abstract

N-methyl-D-aspartate receptor (NMDAR) antibody encephalitis is an autoimmune syndrome with potentially fatal sequelae causing profound dysregulation of neurotransmission. Patients most often present with a constellation of neuropsychiatric signs and symptoms, including behavioral changes, motor disturbances, and seizures. Frequently, the development of anti-NMDAR antibodies has been linked to specific malignancies, although the exact event that triggers the production of these antibodies remains unknown. We present a case of a 25-year-old female who came into the emergency room with behavioral changes and fever. The patient had non-convulsive seizures, catatonia, and orofacial dyskinesias during the course of the admission and was treated as a case of autoimmune anti-NMDAR encephalitis. Cranial and abdominal MRI with contrast initially showed negative results, while the serum and cerebrospinal fluid studies were positive for anti-NMDAR antibodies. The patient was noted to have significant clinical improvement after being treated with high-dose intravenous steroid therapy followed by intravenous immunoglobulin (IVIg) and rituximab infusion. She was discharged stable with the resolution of neurologic symptoms four months after the diagnosis. On follow-up with her neurologist two years later, an abdominal CT scan was done and showed mature cystic teratoma. This is one of the few documented cases of anti-NMDAR encephalitis with a good response to medical treatment but had a delayed diagnosis of ovarian teratoma seen on surveillance work-up years after the diagnosis. A high index of suspicion is warranted for the diagnosis, and treatment should be started early as soon as there is clinical suspicion of the disease. Also, surveillance pelvic or abdominal imaging is important in patients who have negative initial screening but have high risks for teratomas.

## Introduction

Anti-N-methyl-D-aspartate receptor (anti-NMDAR) encephalitis is a form of autoimmune encephalitis that predominantly affects women and is associated with the formation of antibodies targeting the NR1 or NR2 subunits of the NMDA receptor [1]. These receptors are primarily located in the frontotemporal and hippocampal regions, which help explain common psychiatric signs and symptoms, including decreased cognition and personality changes. Patients also commonly exhibit a combination of neuropsychiatric signs and symptoms, such as alterations in behavior, motor disturbances, seizures, auditory and visual hallucinations, delusions, and autonomic disturbances [1,2]. The development of anti-NMDAR antibodies has often been associated with specific malignancies; however, the inciting event that initiates the production of these antibodies remains elusive. Ovarian teratomas were seen in 94% of anti-NMDAR encephalitis patients with neoplasms, and clinical improvements were seen in these patients after tumor removal [2]. The current diagnosis is based on finding anti-NMDAR antibodies in the cerebrospinal fluid (CSF). CSF studies show lymphocytic pleocytosis and normal to mild elevation of protein. Oligoclonal bands may be present in up to 60% of patients. Although there is controversy between testing for serum or CSF antibody titers, CSF titers generally appear to correlate with disease activity [1]. Treatment strategies include resection of the tumor, when applicable, and medical interventions that utilize immune-suppressing agents and other agents that specifically target processes in the pathogenesis of this antibody-mediated disease [3]. ​According to a case series by Dalmau et. al. [4], approximately 75% of patients with NMDA receptor antibodies either experience recovery or exhibit mild sequelae. However, the other 25% had severe deficits or died, with a mortality rate of 7% in 24 months. On the other hand, treatment with first-line immunotherapy resulted in improvement in 53% of patients within the initial four weeks of treatment, 97% of whom showed a good outcome at 24 months [1,3]. Given the high mortality rate, the wide likelihood of presentation, and the potential for treatment, a high index of suspicion and early treatment is warranted for clinicians.

## Case presentation

**Figure 1:**
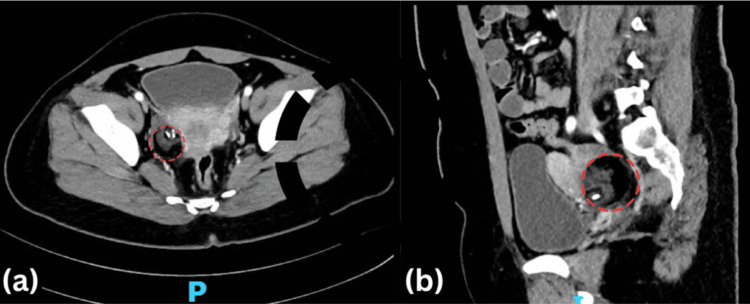
Lower abdominal CT scan (a) Axial view and (b) sagittal view showed a well-defined, fat-attenuating ovarian mass with intralesional calcifications and soft tissue densities measuring 2.6 x 3.2 x 3.9 cm.

The case is a 25-year-old female with no known co-morbidities admitted at a tertiary hospital in the Philippines during the coronavirus 2019 (COVID-19) pandemic for fever and behavioral changes. She initially presented with intermittent low-grade fever two months before admission with no other accompanying symptoms. During the last week of January 2021, the patient was noted to have subtle behavioral changes which manifested as irritability and complaining at work. Three days later, she had intermittent febrile episodes associated with low back pain and body malaise. On the day of admission, the patient was afebrile but had episodes of delusions, restlessness, agitations, and thoughts of persecution. Cranial magnetic resonance imaging (MRI) with contrast showed unremarkable results. She then underwent a lumbar puncture with normal opening and closing pressures. CSF studies showed lymphocyte pleocytosis with normal CSF protein and slightly decreased CSF glucose. Bacterial, fungal, tuberculosis, and viral panels showed negative results. The CSF sample was also sent for the anti-NMDAR panel. During the admission, the patient was noted to have intermittent febrile episodes accompanied by bouts of agitation during the height of the fever. Her two-hour video electroencephalogram (VEEG) showed nonconvulsive status epilepticus. Hence, she was started on valproic acid, levetiracetam, and carbamazepine and was transferred to the intensive care unit (ICU). She then started developing episodes of catatonia accompanied by orofacial dyskinesias during this time. Repeat cranial MRI and abdominal MRI still showed unremarkable results. The patient was then given high-dose methylprednisolone 1 gram/IV for five days. Repeat VEEG done showed a lesser frequency of epileptiform discharges. However, the patient remained obtunded and would occasionally mumble unintelligible words. The patient was then started on IVIg for five days. Afterward, the patient was noted to be more awake, with less orofacial dyskinesias and lower extremity paratonia. The patient was eventually transferred to a regular room and was started on cognitive therapy. Shortly thereafter, CSF and serum anti-NMDAR antibodies returned with a positive result, confirming the diagnosis. Despite clinical improvement, the patient was given one dose of rituximab 500 mg/IV infusion on the second month of hospitalization. Over the subsequent two months, she became increasingly verbal and less agitated, and she was able to tolerate physical therapy. There was no recurrence of orofacial dyskinesias. Antiepileptic drugs were eventually tapered off. Three months into her hospitalization, she still had episodes of confusion and cognitive deficits but was noted to have dramatically improved. The patient was able to tolerate physical therapy. A routine electroencephalogram (EEG) was repeated before discharge with no note of epileptiform discharges. The patient was discharged awake, coherent, and ambulatory with no involuntary movements or seizure episodes. She had regular follow-ups with her attending neurologist and was able to return to her work as a nurse in a tertiary hospital. An abdominal computed tomography (CT) scan was done as surveillance two years after hospitalization which showed an incidental finding of a mature cystic teratoma (Figure 1). Although clinically the patient did not have any signs of active encephalitis, the patient underwent a laparoscopic right oophorectomy which she tolerated well. Surgical pathology confirmed the diagnosis of a mature cystic teratoma on the right. Currently, the patient has no involuntary movements or behavioral changes. She was able to continue activities of daily living independently and is back to work.

## Discussion

Anti-NMDAR encephalitis is a form of autoimmune encephalitis associated with the formation of CSF immunoglobulin G (IgG) antibodies against the NR1 and NR2 subunits of the NMDA receptors in the central nervous system (CNS). This causes subsequent internalization of NMDA (glutamate) receptors, causing a reduction of neuronal calcium influx and a decrease in the receptor-dependent synaptic currents which are responsible for most of its symptoms [4-6]. Group of symptoms develop and progress according to stages of disease in anti-NMDAR encephalitis. Initially, a prodromal illness is followed by psychiatric manifestations and prominent neurologic manifestations, including movement disorders, seizures, autonomic dysfunction, and impaired consciousness. Lastly, there is a long recovery stage with persistent neurologic symptoms [3]. This disease is estimated to occur at a rate of 1.5 cases per million per year, exhibiting a higher frequency in childhood and having a median onset age of 21 years. This disease most commonly affects young adult females aged 25 to 35 years, with a female-to-male ratio of 4:1 [4,5]. The presence of an ovarian teratoma triggers antibody production in 85%-94% of cases, with rare instances involving other tumors. Nevertheless, the precise cause of antibody production remains unknown in the majority of cases [2].

Treatment for suspected autoimmune encephalitis is often given empirically before specific antibody test results. Identification of NMDAR antibodies confirms the diagnosis of the disorder and should trigger the exploration of a tumor [7]. Half of the cases of anti-NMDA receptor encephalitis among females in the reproductive years are linked to an ovarian teratoma, which can be microscopic. Patients older than 18 years, particularly females of Asian or African-American descent, have higher risks of developing ovarian teratomas. Hence, every imaging abnormality in these patients should be thoroughly considered [3,8]. In addition, even small or microscopic ovarian teratomas containing nervous tissue may express NMDAR subunits that interact with the patient’s antibodies primarily affecting the hippocampus and forebrain regions and leading to a cascade of symptoms causing anti-NMDAR encephalitis. Thus, in these instances, tumor resection and immunotherapy result in improvement or full recovery [9]. As there is no serum tumor marker for ovarian teratomas, the recommended screening includes ultrasound and pelvic MRI. A good prognosis is observed when there is timely identification and prompt removal of an ovarian teratoma. The best management for patients exhibiting a high clinical likelihood of an ovarian teratoma but with negative initial imaging studies remains unclear. Options for these patients include initiating immunotherapy without additional investigation for ovarian teratoma, conducting periodic screenings every six months for four years to detect subtle abnormalities indicative of a microscopic teratoma, and performing exploratory laparoscopy or blind oophorectomy [3,8]. Some authors recommended proceeding with exploratory laparotomy and large wedge resection of the ovary in patients with negative pelvic or abdominal imaging tests to increase the likelihood of identification of a microscopic teratoma and to improve outcomes, especially in patients who are refractory to immunotherapy and have anti-NMDAR antibodies, but this issue remains controversial [9]. Furthermore, the possibility of neoplasms should be consistently taken into account during both the initial treatment and follow-up visits. This consideration is important not only for identifying serious cancers but also because specific tumors may indicate particular autoimmune causes [10].

The mainstay of treatment generally involves an escalation of immunotherapy alongside teratoma removal, if applicable. Early initiation of immunotherapies has demonstrated efficacy in improving outcomes and minimizing relapses. During the acute phase of the disease, initial immunotherapy consists of a combination of high-dose steroids, immunoglobulins, and/or plasma exchange. When first-line therapy fails, giving second-line immunotherapy, particularly rituximab, can further improve outcomes and prevent relapses. The likelihood of relapse decreases when first-line immunotherapy is initiated in the acute phase of the disease, and this risk is further diminished with the use of second-line immunotherapy during the relapse stage [3,8]. Factors linked to favorable outcomes in patients with autoimmune anti-NMDAR encephalitis include prompt and early initiation of treatment, no need for ICU support, and a low disease severity within the initial four weeks of presentation [10].

## Conclusions

This case is one of the few documented case reports of anti-NMDAR encephalitis exhibiting a favorable response to medical treatment but had a delayed diagnosis of ovarian teratoma seen on surveillance work-up years after the diagnosis. With this, we aim to highlight anti-NMDAR autoimmune encephalitis as an important diagnostic consideration in patients presenting with distinct psychiatric features who eventually evolved to develop neurologic findings such as orofacial dyskinesias and catatonia. A high index of suspicion is important to diagnose the disease, and treatment should be started as soon as there is a clinical assessment for the disease. Our case report also highlights the importance of remaining vigilant for an underlying ovarian teratoma and the importance of surveillance abdominal or pelvic imaging in patients with negative initial screening but with a high likelihood of a gynecologic neoplasm. If a tumor is found, prompt removal is important since this hastens improvement and decreases the risk of relapses.
